# Role of chemokines in ectopic lymphoid structures formation in autoimmunity and cancer

**DOI:** 10.1002/JLB.3MR0218-062R

**Published:** 2018-06-27

**Authors:** Alessandra Nerviani, Costantino Pitzalis

**Affiliations:** ^1^ Centre for Experimental Medicine & Rheumatology, William Harvey Research Institute, Barts and The London School of Medicine and Dentistry Queen Mary University of London London UK

**Keywords:** chemokines, ectopic lymphoid structures, rheumatoid arthritis, synovium

## Abstract

Ectopic (or tertiary) lymphoid structures (ELS) are organized aggregates of lymphocytes resembling secondary lymphoid organs and developing in chronically inflamed nonlymphoid tissues during persistent infections, graft rejection, autoimmune conditions, and cancer. In this review, we will first depict the mechanisms regulating ELS generation, focusing on the role played by lymphoid chemokines. We will then characterize ELS forming in target organs during autoimmune conditions, here exemplified by rheumatoid arthritis, and cancer, highlighting the relevance of the tissue‐specific factors. Finally, we will discuss the clinical significance of ELS and the therapeutic potential of their inhibition and/or enhancement depending on the disease considered.

## Abstract 2

Review of how lymphoid chemokines regulate the ectopic development of lymphoid structures in ‘permissive’ tissues during chronic inflammation.

**Abbreviations**
APRIL: a‐proliferation‐inducing ligand
C: cysteine
CCP: cyclic citrullinated protein
DCs: dendritic cells
ELN: ectopic lymphoid neogenesis
ELS: ectopic lymphoid structures
FDCs: follicular dendritic cells
GCs: germinal centers
HEVs: high endothelial venules
ILC: innate lymphoid cells
KO: knockout
LT: lymphotoxin
LTi: lymphoid tissue inducer/initiators
LTo: lymphoid tissue organizer
PNAd: peripheral nodal addressin
RA: rheumatoid arthritis
SLOs: secondary lymphoid organs
Tfh: T follicular helper
TLOs: tertiary lymphoid organs
Treg: T regulatory.

## 1 INTRODUCTION

Chemokines are small (7–12 kDa) chemotactic polypeptides sharing a common structural motif^1^ and able to direct lymphocyte recruitment and organize the architecture of lymphoid organs in health and disease.^2^ Chemokines’ structure is characterized by four conserved cysteine residues (C). When the first two CC are sequential, chemokines are defined CCL; conversely, if the CC sequence is divided by a single amino acid (X), they are labeled CXCL.^3^ More recently, another unique subfamily of chemokines has been discovered and named CX3C. This includes only one member (fractalkine or CX3CL1), which, atypically, can exist in either a membrane‐bound or a soluble form.^4^

Chemokines are produced by multiple cell subsets and can be categorized according to the nature of the expressing cells. Homeostatic chemokines, for example, CXCL12, CXCL13, CCL19, and CCL21 are constitutively expressed in lymphoid organs; inflammatory chemokines such as CXCL1, CXCL2, and CXCL3 are typically produced in response to inflammation.^5^

Both lymphocyte migration and segregation of B and T cells in their characteristic microcompartments in lymphoid organs depend on the interaction between chemokines and their G‐protein coupled receptors,^2^ for example, CXCL12 binding CXCR4, CXCL13 binding CXCR5, CCL19 and CCL21 binding CCR7.^2^

Lymphoid organs are highly differentiated compartments responsible for coordinating and guiding the differentiation and proliferation of lymphocytes, hence directing immunologic responses in all their complexity. Bone marrow and thymus, defined as *primary* lymphoid organs, are the anatomic sites dedicated to the selection and maturation of naïve B and T lymphocytes from immature hematopoietic precursors. *Secondary* lymphoid organs (SLOs) coordinate adaptive immunologic responses through the trafficking of lymphocytes from peripheral sites following antigen (Ag) encounter^6^ via afferent lymphatics and maintain immune tolerance to auto‐Ag.^7^ SLOs include spleen, lymph nodes, and mucosal‐associated lymphoid tissue (MALT) such as Peyer's patches and tonsil.

Chronically inflamed nonlymphoid tissues can also host highly organized aggregates of lymphocytes known as *tertiary* lymphoid organs (TLOs) or ectopic lymphoid structures (ELS). These structures usually occur in the course of persistent infections, transplant rejection, cancer (reviewed in Ref. 8), and autoimmune diseases (reviewed in Ref. 9), in response to mediators of inflammation like chemokines, cytokines, and bioactive lipids produced by tissue‐resident cells and able to regulate the recruitment and organization of lymphocytes. In some circumstances, ELS acquire the name related to the anatomic district in which they develop, for instance, iBALT (inducible bronchus‐associated lymphoid tissue) in the lungs.^10^ The process responsible for the development of ELS is called ectopic lymphoid neogenesis (ELN) and, differently from primary and secondary lymphoid organogenesis, it occurs after birth and is not genetically programmed.^11^

ELS are dynamic structures resembling the cellular arrangement of SLO. Although with varying degrees of organization, ELS are typically characterized by (i) a distinct T‐lymphocytes rich zone enclosing a central B‐cell rich area; (ii) a network of follicular dendritic cells (FDCs) and activated stromal mesenchymal cells (e.g., lymphoid tissue fibroblasts)^12^, ^13^; (iii) plasmablasts and plasma cells surrounding the T cell rich area; and (iv) high endothelial venules (HEVs), which are postcapillary blood vessels normally not found in peripheral tissue but typical of SLOs and dedicated to favoring the migration of naïve lymphocytes into SLOs.

ELS share the genetic profile of SLOs, including the expression of genes encoding lymphoid chemokines and lymphotoxins (LTs), and often contain functionally active germinal centers (GCs) able to mediate in situ B cell differentiation, somatic hypermutation, oligoclonal expansion and, eventually, antibodies production. The critical factors driving ectopic neogenesis of lymphoid structures in peripheral diseased tissues, including lymphoid chemokines, overlap substantially with the molecular machinery supporting SLOs’ prenatal development.^14^ Nevertheless, the cellular components producing these key modulators may differ in ELS. A few architectural dissimilarities between ELS and SLOs also exist: while the SLOs are enclosed by a fibrous capsule and have an independent afferent lymphatic vessel (except for MALT), ELS lack both, form deeply within the connective tissue and are exposed directly to Ag and regulatory molecules produced within the inflamed tissue.^15^ Such microarchitectural differences of ELS, in the case of persistent infections, could improve the immunologic response, enhancing the production of antibodies directed against the pathogenic microorganisms. Similarly, several findings also support a beneficial role for ELS in cancer. Conversely, in tissues target of autoimmune processes, the constitutive exposure to the auto‐Ag not only can favor the development of ELS but also, in turn, expand the autoreactive response with the proliferation of autoreactive T and B cells and increase the local production of autoantibodies.^16^

Here, we will initially describe the regulatory mechanisms of the ELS generation, using SLOs as a comparator and focusing on the role played by lymphoid chemokines. We will then define peculiar features acquired by ELS when forming in target organs in the course of certain pathologic conditions, specifically rheumatoid arthritis (RA) and cancer.

## 2 REGULATORY MECHANISMS OF THE ELN: THE KEY ROLE OF HOMEOSTATIC CHEMOKINES

The development of both SLOs and ELS is a rather sophisticated and finely regulated mechanism, which is largely orchestrated by lymphoid chemokines, cytokines, adhesion molecules, and survival factors.^17^

During the embryonic life, the early phase of the secondary lymphoid organogenesis involves the crosstalk between the hematopoietic‐derived CD3^−^CD4^+^IL‐7Ra^+^RANK^+^ and/or CD3^−^CD4^−^CD45^+^IL‐7Ra^−^RANK^+^CD11c^+^CD11b^+^ lymphoid tissue inducer/initiators (LTi) cells and the mesenchymal lymphoid tissue organizer (LTo) VCAM‐1^+^ICAM‐1^+^LTβR^+^ cells.^11^, ^18^ The interaction between LT α1β2 (LTβ), produced by LTi, and its receptor LTβR, expressed by LTo cells, initiates the secondary events of the SLOs generation.^15^ These take place in the presence of IL‐7 and RANK‐ligand and consist of the recruitment and the retention of lymphocytes. The former is determined by the high gradient of CXCL13, CCL19, and CCL21 produced by LTo cells in response to LTβ, whereas the latter occurs through the up‐regulation of the adhesion molecules (VCAM1, ICAM1) and peripheral nodal addressin (PNAd) in HEVs and stromal cells.^11^, ^19^ The expression of CXCR5 and CCR7 on the surface of LTi confers to these cells the ability to respond to CXCL13 and CCL19/CCL21, respectively. Eventually, LTβ/LTβ‐R, CXCL13/CXCR5,^11^ and CCL19/CCL21/CCR7^20^ sustain the recruitment and segregation of B/T cells in distinct areas.^21^

The chief role of the CXCL13/CXCR5 during the lymphoid organogenesis has been demonstrated in animal models in which this axis was either silenced or overexpressed. Mice deficient for CXCL13/CXCR5 showed an incomplete maturation of the lymph nodes, with some of them formed and some others missing.^22^ The overexpression of this pathway prompted the LTβ‐dependent development of ELS in nonlymphoid organs.^21^

Along with the CXCL13/CXCR5 axis, also CCR7, the common receptor for CCL19 and CCL21, plays a relevant role in the initial phase of the lymphoid organogenesis.^20^ Mice deficient for CCR7 have, at birth, almost all the SLOs as their wild‐type counterparts, though the lymphocytes segregation in these organs is impaired and the architecture altered.^23^ Overall, the effect of CCR7 and CXCR5 and their cognate ligands is synergic; in fact, in CCR7/CXCR5‐double knockout (KO) mice the number of undeveloped lymph nodes is higher compared with the single KO for each of these chemokine receptors.^24^

Conversely to SLOs, the *primum movens* of the ELS generation in peripheral nonlymphoid adult tissues has not been entirely elucidated yet. Specific inflammatory signals and the cellular microenvironment of the tissue are critical elements, as suggested by the preferential development of the ELS in particular tissue/organs (“permissive tissues”) and in certain but not all patients.^25^ For example, the overexpression of the homeostatic chemokine CCL21 in animal models is sufficient for inducing ELN in the pancreas but not in the skin.^26^

Similarly to SLOs, the immune cells infiltrating the target tissue during the inflammatory process behave as inducers of ELS^27^; on the other hand, the resident stromal cells mirror the activity of the mesenchymal LTo as seen during the secondary lymphoid organogenesis.^12^, ^28^, ^29^ Stromal cells include fibroblasts, endothelial and epithelial cells, and pericytes.^30^

As it happens in SLOs, homeostatic lymphoid chemokines are essential for the correct clustering of B/T cells and the development of adequately arranged lymphoid structures.^14^, ^31^ Nevertheless, in nonlymphoid organs, the ectopic generation of lymphoid follicles may require additional signals provided by the chronically inflamed tissue, for example, persistent Ag presentation.^17^

With regards to the infiltrating immune cells acting as inducers, the development of ELS has been shown to be strongly dependent on the presence of the Th17 subset of cells and its eponymous cytokine IL‐17 in animal models of lung inflammation, multiple sclerosis, and inflammatory arthritis.^32^, ^33^ Specifically, the expression of the glycoprotein podoplanin seems to be critical.^27^ The migration and retention of Th17 cells is likely related related to the CCL20/CCR6 axis. In fact, Th17, alongside with immature dendritic cells (DCs), express CCR6,^17^ which represents the cognate receptor of the chemokine CCL20. Studies characterizing tertiary lymphoid structures forming in lungs during idiopathic/heritable pulmonary arterial hypertension demonstrated high levels of CCL20 within the ELS, and a substantial infiltration of CCR6^+^IL17^+^ T cells CCL20‐sustained.^34^ IL‐17, however, is not the only cytokine involved: in fact, also IL‐23 and IL‐22, respectively upstream and downstream of the Th17 signaling, promote ELN in target organs, as observed in rheumatoid synovial tissue^35^ and salivary glands in experimental Sjogren's syndrome.^36^

It has been recently described that a group of innate lymphoid cells (ILC), probably ancestrally linked to the Th17 cells, and called “adult LTi”^37^, ^38^ can also contribute to the development of ectopic lymphoid tissue. This process occurs by exploiting the same downstream pathway used by the Th17 subset.^33^, ^39^ Furthermore, it has lately emerged that also IL‐21‐producing and ICOS‐expressing T follicular helper (Tfh) cells may be involved in ELS generation and activities, as the organization of the GC and the production of high‐affinity immunoglobulins appear deranged in the absence of Tfh.^40^ In line with that, an increased rate of circulating Tfh cells can be detected in several autoimmune conditions characterised by ELS formation.^41^ Notably, some features typical of Tfh, for example, IL‐21 and ICOS production/expression, can also be acquired by Th17 cells during their differentiation.^8^, ^42^, ^43^, ^44^

If Th17 cells, adult LTi‐Th17‐like cells and Tfh cells are contributors to ELS, a growing body of evidence has instead suggested that immunosuppressive Foxp3^+^ T regulatory (Treg) cells may act as negative regulators of the ELN, in particular by preventing the development of HEVs.^45^ T cell subsets are not the only immune cells able to induce TLOs neogenesis; inflammatory macrophages (M1‐polarized) have been indeed described to be able to stimulate vascular smooth muscle cells to express homeostatic chemokines which, eventually, lead to ELS formation.^46^

Overall, the cytokines released by the immune cells acting as inducers of ELS prompt the production of essential lymphoid chemokines such as CXCL13 and CXCL12 which, in turn, promote the recruitment of naïve B cells. The chemotactic gradient of CXCL13 constitutes a potent homing signal for the CXCR5^+^ B lymphocytes. Once recruited into the follicle, B cells further sustain ELS formation by producing LTβ. Tfh cells not only participate as potential inducers of ELS but they underpin the biologic activity of B cells, including the differentiation into antibodies producing cells within the GC.^47^ Tfh cells express CXCR5, which gives them the ability to respond to CXCL13 and locate in the proximity of B cells in ELS, thus providing the environment for Ag‐specific B cells help. Remarkably, Tfh cells become themselves producers of CXCL13 contributing to the formation of second immunologic synapses.^48^, ^49^

Though in TLOs the micro‐anatomic organization in dark and light zone is not as well defined as in SLOs, the functionality of local antibodies production relies on the shuttling of B‐lymphocytes between the equivalent of the dark and light zone. GC B cells accumulate at the site of the Ag selection in the light zone following CXCL13 chemo‐attraction, whereas centroblasts expressing CXCR4 are recruited in the dark zone, site of somatic hypermutation, in response to CXCL12 predominantly released by the tingible body macrophages.^28^ CXCR5^+^‐Tfh cells, attracted by CXCL13, are recruited inside the follicle where they can contribute to the establishment of the GC.^50^ Vice versa, high levels of CCL19 and CCL21 keep CCR7^+^ un‐primed T cells towards the periphery, outside the follicle.

Although its role is yet to be fully defined, the unique chemokine CX3CL1 seems to be likewise involved in the generation of ELS in autoimmune diseases (i.e., in salivary glands of Sjogren's syndrome), possibly by attracting CX3CR1^+^‐precursors of resident DCs.^51^

A primary role in the lymphocytes recruitment and retention in both SLOs and ELS is played by the HEVs. These are peculiar vascular structures characteristic of lymphoid organs. During secondary lymphoid organogenesis, HEVs are generated in response to factors released by LTo, for example, fibroblast growth factor‐2.^11^ In nonlymphoid peripheral inflamed tissue, HEVs seem to become PNAd‐expressing upon the initial interaction between T and DC cells surrounding the blood vessels.^52^

The diapedesis and the homing of naïve T cells to SLOs and TLOs require the initial interaction between PNAd, expressed by the high endothelial cells in HEVs, and its ligand L‐selectin/CD62L, expressed by lymphocytes.^53^, ^54^ Subsequently, CCR7‐expressing T cells are attracted towards their cognate ligands CCL19/CCL21, which are produced on the abluminal side of the HEVs in the T cell area.^20^

PNAd+ endothelial cells themselves are an additional source of the CCR7‐ligand CCL21 in order to be posted on the luminal surface and initiate the adhesion cascade and the transmigration of CCR7^+^ L‐selectin^+^ Naïve T cells.^52^, ^55^ Mature DCs, which are CCR7^+^ cells, similarly migrate to the SLOs/TLOs following CCL19/CCL21 gradient. B cells can express CCR7 on their surface too, yet they use it only during the adhesion phase to the endothelium of HEVs since the transmigration to the follicle is primarily guided by CXCR5/CXCL13 and CXCR4/CXCL12.^20^

Interestingly, while the role of CCR7 in secondary lymphoid organogenesis is well defined and the features of SLOs in CCR7^−^/^−^ are consistent with its ability to organize the architecture of the follicle, the CCR7 involvement and function in tertiary lymphoid structures formation are instead rather more ambiguous. On the one hand, CCR7^−^/^−^ mice unexpectedly showed the presence of ELS at various mucosal sites, for example, salivary glands.^56^ On the other hand, in a model of chronic Ag‐induced arthritis, deleting CCR7 could inhibit ELS development.^57^ Furthermore, CCL21 overexpressed in nonlymphoid organs (liver, pancreas) can induce the development of ectopic SLOs‐like structures.^20^, ^26^ In trying to resolve the controversy, it is worth bearing in mind that the global phenotype of the CCR7^−^/^−^ model is characterised by an impairment of the T lymphocytes negative selection, the T cells migration and, importantly, the Treg correct functioning. In this model, lymphocytes can unrestrainedly penetrate different organs and there, in the absence of properly functional Treg cells, arrange as ectopic lymphoid follicles in a CCR7‐independent way, as opposed to the CCR7‐dependent manner occurring during the ELS‐development secondary to CCR7‐ligands overexpression.^20^, ^58^

Treg cells, however, not only act as negative regulators of ELS generation but can also be present inside the ELS where they might play antithetic roles. In fact, if the presence of intra‐ELS Treg in neoplastic diseases usually associates with the suppression of the host response against cancer cells,^59^ vice versa, in a model of atherosclerosis, the infiltration of anti‐inflammatory activated Treg cells skews the immune response towards an “anti‐atherogenic” phenotype, rendering aortic TLOs protective.^60^, ^61^

Overall, even if ELS forming in chronically inflamed tissues are essentially induced and maintained by the same chemokines and LTs regulating SLOs’ development, these molecules can be produced at the site of the disease by additional or alternative sources, often represented by subsets of tissue‐specific cells. In SLOs, the foremost source of CXCL13 in the GC is the network of FDCs, which originate from LTo and support the affinity maturation of the B cells.^31^, ^62^ During inflammation also different T cell subsets (e.g., memory T cells,^63^ Tfh,^49^ T peripheral helper [Tph] cells^64^) and activated monocytes/macrophages can become CXCL13 producers.^65^ Moreover, also activated stromal components such as epithelial cells and myofibroblast‐like cells can release CXCL13^66^ and CCL21,^67^ respectively.

Alongside with chemokines, also LTs are critically important for TLO neogenesis, both in cancer and chronically inflamed tissues.^45^

Lymphoid chemokines and LTs work in concert with numerous cytokines to shape the cellular microenvironment during tertiary lymphoid neogenesis. In that context, in addition to the importance mentioned above of the Th17‐related cytokines (IL‐17, IL‐22, IL‐23) and the Tfh‐produced IL‐21, available data suggest that a number of other “positive regulators” contribute to ELS formation. For example, IL‐36 agonists, members of the IL‐1 sub‐family of cytokines together with IL‐1α, IL‐1β, IL‐18, and IL‐33,^68^ have indeed recently emerged as novel promoters of ELS generation in inflamed and neoplastic tissues.^69^ This effect likely depends on the IL‐36‐mediated ability to induce proinflammatory cytokines and chemokines that in turn recruit B/T lymphocytes^69^ and differentiate Th1 and IL‐17‐producing T cells.^68^

Recent studies have also identified a number of “negative regulators” of ELS. For example, IL‐27 has emerged as a negative regulator of the Th17‐mediated generation of ELS in synovia during inflammatory arthritis.^27^

In summary, it is evident that lymphoid chemokines undoubtedly play a vital role in the lymphoid organogenesis and are essential for the initiation and maintenance of the ELS. The features and relevance of tissue‐specific factors in ELS development in the context of autoimmunity and cancer will be discussed in the next section.

## 3 ELN IN DISEASES: THE RELEVANCE OF TISSUE‐SPECIFIC FACTORS

As discussed above, the importance of lymphoid chemokines in the development of ELS is widely accepted. These structures can be detected in target organs of several pathologic conditions in which they play diversified roles. In autoimmune diseases, ELS become microniches of autoreactive activated B cells and plasma cells and likely contributors to the disease pathogenesis and chronicity. Conversely, in persistent infective diseases, ELS might help to confine the immune reaction to the infected site. Therefore, ELS can improve the resolution of the infection. However, by the same token, their presence may also increase the risk of developing autoimmunity through molecular mimicry. In cancer instead, ELS appear to be able to enhance the antineoplastic activity of the immune system. Thus, in the final section of this review, we will discuss the features of ELS in specific pathologic contexts.

## 4 AUTOIMMUNE DISEASES: THE PARADIGM OF RA

A growing body of evidence has confirmed that ELS characterize target organs in numerous autoimmune conditions, including Hashimoto thyroiditis, myasthenia gravis, type I diabetes, multiple sclerosis, Sjogren's syndrome,^70^ and RA.^9^, ^71^ In this setting, ELS are chronically triggered and possibly contribute to sustaining the pathogenic process^8^ and the local production of autoreactive antibodies.^72^

The introduction of minimally invasive techniques of synovial tissue sampling^73^ has empowered the in‐depth analysis of the diseased synovium. Several data about the incidence of ELS and the mechanisms regulating their development in RA have become available since then, representing a valuable paradigm of how ectopic lymphoid structures form and function in autoimmune diseases. However, not all patients affected by RA develop ELS at the site of the inflammation. ELS can be detected in the synovial tissues of around 40% of patients, but only 10–25% of these structures acquire features of fully formed TLO including the presence of a functional GC.^25^

The presence of a lymphoid‐like‐synovitis seems to define a subset of patients with increased disease activity,^74^ more prone to early bone erosions (our unpublished data) and particularly difficult to treat.^75^, ^76^ The factors driving ELN in RA include the local up‐regulation of the classic B lymphoid chemokines CXCL13 and CXCL12,^31^, ^62^, ^67^, ^77^, ^78^ the production of the BAFF and the a‐proliferation‐inducing ligand (APRIL) by fibroblasts‐like‐synoviocytes,^79^ the release of CCL21 by myofibroblast‐like stromal cells^67^ and of CCL20 by activated synovial fibroblasts and osteoblasts.^80^, ^81^

In the highly inflamed microenvironment of the rheumatoid synovia, activated mesenchymal cells become efficient lymphoid‐tissue organizer cells, able to produce chemokines and cytokines that, in turn, favor ELS generation.^77^ To this extent, also the release of cytokines from infiltrating CCR6^+^ Th17, attracted by CCL20,^81^ and Tfh cells gives a further contribution.^27^ As in SLOs, CXCL13 is produced by FDC within the GC. However, other CXCL13‐producing cells have been described in the inflamed synovia, for example, a subset of Ag‐experienced Th cells originally detected by Manzo et al.^63^ More recently, a subset of PD1^+^ CXCR5^+^ CD4 T cells named Tph cells has been characterized to be significantly expanded in the rheumatoid synovitis, where it may provide a prompting signal to the development of ELS by producing CXCL13/IL‐21 and recruiting B cells/Tfh.^64^ In keeping with the importance of Tfh, circulating levels of IL‐21 seem to correlate with anti‐cyclic citrullinated protein (CCP) antibodies and the severity of RA.^82^ Silencing the IL‐21 pathway instead improves the clinical outcome in experimental arthritis models.^83^ Genetic analyses of lymphoid‐like RA synovial tissue have confirmed the local up‐regulation of lymphoid‐associated genes, for example, IL‐7, one of the crucial players of the SLO development,^84^ the IL‐21/IL‐21R axis,^27^ and the full set of homeostatic chemokines CXCL13/CCL19/CCL21.^16^, ^62^, ^72^, ^85^ Among them, it has been reported that levels of circulating CXCL13 positively correlate with the local expression of CXCL13 within the rheumatoid synovial tissue^86^ and are significantly higher in patients characterized by a synovial lymphoid transcriptomic profile.^85^ Serum CXCL13 has been also proposed as a biomarker of response to targeted biologic treatments. It seems indeed that patients with baseline high levels of CXCL13 in association with low concentrations of the myeloid marker soluble ICAM1 are more likely to respond to anti‐IL‐6R agents. Vice versa, a serum profiling characterized by low CXCL13/high ICAM1 associates with higher rates of response to the TNFα blockade.^85^

This is not surprising as TNFα itself might contribute to synovial ELN too. In fact, the ELS reversal observed in the synovial tissue of some RA patients following the inhibition of TNFα by specific blocking agents would indirectly imply that TNFα is itself a contributor to ectopic lymphoid synovial neogenesis^75^

**Figure 1:**
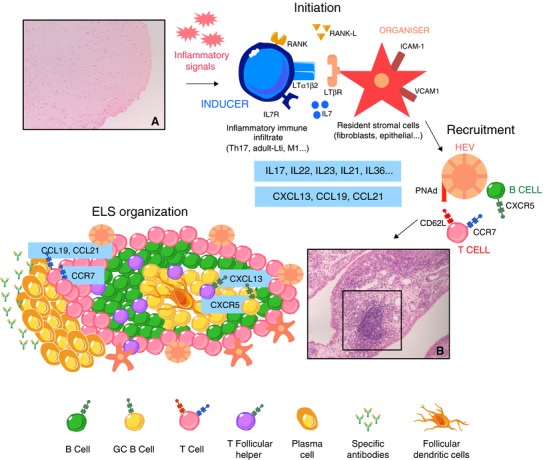
**Ectopic lymphoid structures (ELS) generation in the inflamed synovial tissue of patients affected by rheumatoid arthritis (RA)**. Uninflamed synovium shows scant infiltration of immune cells [A, H&E staining]. [Initiation] In response to chronic inflammatory signals characterizing RA synovitis, several cell types (e.g., adult LTi, Th17, M1 macrophages), attracted by homeostatic cytokines (CXCL13, CCL21), behave as initiators of the ectopic lymphoid neogenesis. Resident stromal cells such as fibroblasts‐like‐synoviocytes and myofibroblast‐like cells act as LTo, contribute to lymphoid chemokines production, and guide the cellular arrangement of the follicle‐like‐structure. [Recruitment] In their initial phase, ELS are characterized by the development of HEVs, which are of fundamental importance for enhancing T and B cells recruitment to the site of inflammation. [ELS organization] Once ELS are formed within the synovial tissue [B, H&E staining], both Ag‐presenting FDCs and B/Tfh cells shuttling into the GC help maintaining these structures. Different gradients of CXCL13 and CCL19/CCL21 support the segregation of B/T cells. Eventually, B cells differentiate in situ into plasma cells, which produce disease‐specific anti‐CCP autoantibodies. CCL, chemokine (C‐C motif) ligand; CCR, chemokine (C‐C motif) receptor; CCP, cyclic citrullinated protein; CXCL, chemokine (C‐X‐C motif) ligand; CXCR, chemokine (C‐X‐C motif) receptor; ELS, ectopic lymphoid structures; FDCs, follicular dendritic cells; GC, germinal center; HEVs, high endothelial venules; ICAM1, intercellular adhesion molecule 1; LTβR, lymphotoxin‐β receptor; LTi, lymphoid tissue inducer; LTo, lymphoid tissue organizer; PNAd, peripheral nodal addressin; RA, rheumatoid arthritis; RANK, receptor activator of NF‐κB; RANKL, RANK ligand; Tfh, T follicular helper; Th, T helper.

As the availability of the homeostatic chemokines increases within the synovium, the cellular arrangement of the ELS becomes increasingly more “organised,”^25^, ^74^ eventually enabling the development of a functionally active GC, which support the occurrence of activation‐induced cytidine deaminase‐dependent class‐switching and somatic hypermutation with the production of high affinity, RA‐specific anti‐CCP antibodies.^72^ The crucial elements and steps of the ELS generation in RA synovial tissue have been depicted in Fig. 1.

## 5 CANCER: NEW INSIGHTS

Over the last decade, a huge effort has been made to identify and potentially manipulate for therapeutic purposes the mechanisms controlling the ELN in cancer.^45^, ^87^

Similarly to autoimmune diseases, the molecular machinery inducing the intra/peritumor ELN seems to largely overlap with the generation of SLO, including the up‐regulation of the homeostatic chemokines CXCL13, CCL19, and CCL21.^88^

For example, studies in lung carcinoma have confirmed that CXCL13 is produced by FDCs located in the GC‐like zone and constitutes the chemotactic signal for CXCR5^+^ Tfh, which can be detected in the same area of FDCs. CCL21, instead, is predominantly produced in the lymphatic vessels.^45^ Overall, a more favorable outcome has been observed in patients with solid cancers, for example, breast cancer,^89^ colorectal carcinomam,^90^ melanoma,^91^ and non‐small cell lung cancer^92^ characterized by the presence of ELS within the neoplastic tissue.^69^

In particular, it has been described that the infiltration of CXCL13‐producing Tfh cells within the intratumor tertiary lymphoid structures associates to a better clinical outcome in patients with breast cancer.^43^, ^93^ A high CXCL13 signature has also been demonstrated to be a convincing marker of better prognosis in ovarian and colon cancer.^94^ Consistently, an improved antitumor immune response has been associated with the presence of highly organized lymphoid structures including HEVs in both animal models and patients affected by colorectal carcinoma.^95^ In keeping with the negative regulatory effect of Treg cells on ELS development, it has also been shown that the ablation of this subset during experimental carcinogenesis methylcholanthrene‐induced correlated with the formation of lymphoid aggregates. Remarkably, a better clinical outcome was achieved when the ELS were properly organized and included HEV.^96^

The crucial importance of HEVs has been confirmed in multiple studies, in which the presence of this peculiar ELS‐associated vasculature structure correlates with better survival rates and decreased incidence of metastasis.^45^

Therefore, the possibility of “controlling” the development of intratumor ELS to improve the immune reaction to cancer cells represents an attractive therapeutic option. With this intention, current studies have tried to delineate the set of chemokines mainly responsible for cancer‐related ELN, eventually identifying a 12‐chemokines signature able to precisely predict the features of the ELS forming at the site of a tumor.^91^ Importantly, the definition of the best candidates able to induce ELN needs to take into account the considerably immunosuppressive environment characterizing neoplastic processes.^87^ Nevertheless, the clinical success of checkpoint inhibitors in the treatment of solid tumors has validated the approach of modulating natural immune responses as anticancer therapy.^97^, ^98^

## 6 CONCLUDING REMARKS

The critical role of lymphoid chemokines in shaping ectopic neoformed SLOs‐like structures has been widely demonstrated. The significance and functional outcome of these structures, however, can be extremely different. In autoimmune diseases, here exemplified by RA, ELS seem to actively contribute to the maintenance of the immune response at the site of inflammation, hence participating in the local development of autoimmunity and tissue damage. On the contrary, intratumor tertiary lymphoid structures driven by homeostatic chemokines appear to be associated with a more favorable prognosis.

Further studies aiming at outlining the context‐specific mechanisms of the ELN will help shed light and improve the clinical targeting.

In fact, depending on the pathologic process, the therapeutic approaches to target ELS may hugely vary. On the one hand, agents inhibiting lymphoid chemokines, LTs and adhesion molecules might be exploited to contain the persistent immune response in autoimmune conditions. Conversely, similarly to the success of checkpoint inhibitors in solid tumors, enhancing immune responses through the development of ELS via inducing lymphoid chemokines might represent a novel therapeutic approach for treating cancers.
